# A Case of Clinically Suspected Epidermolysis Bullosa With Oesophageal Stricture Successfully Managed by Savary–Gilliard Dilatation

**DOI:** 10.1155/crgm/1227580

**Published:** 2026-09-15

**Authors:** Mohammad Abu Faisal, Sara Tasnim Mahpara

**Affiliations:** ^1^ Department of Gastroenterology, Chittagong Medical College, Chattogram, Bangladesh, cmc.edu.bd; ^2^ Department of Gastroenterology, Chittagong Medical College and Hospital, Chattogram, Bangladesh

**Keywords:** bullous skin lesions, dysphagia, epidermolysis bullosa, esophageal stricture, hypopigmented scars

## Abstract

**Background:**

Epidermolysis bullosa (EB) comprises a heterogeneous group of inherited blistering disorders characterized by skin and mucosal fragility. Oesophageal involvement, though uncommon, is a recognized and potentially debilitating complication.

**Case Presentation:**

A 10‐year‐old girl presented with progressive dysphagia to solid foods of one‐and‐a‐half months duration, against a lifelong history of recurrent bullous skin lesions healing with scarring and nail dystrophy; a younger sibling had similar skin lesions. Upper gastrointestinal endoscopy revealed oesophageal strictures from 18 cm to 22 cm from the incisors (combined length approximately 5 cm), which could not be traversed by the scope. Barium swallow confirmed a tight upper‐thoracic stricture with pre‐ and poststenotic dilatation. Skin biopsy showed a subepidermal vesiculobullous disorder with eosinophils, with direct immunofluorescence negative for IgA, IgG, and C3; genetic testing, immunomapping, and electron microscopy were unavailable. Based on the lifelong cutaneous phenotype, family history and histopathological findings, a diagnosis of clinically suspected inherited EB was made, with the oesophageal stricture considered to be occurring in this context. The patient underwent fluoroscopy‐guided, guidewire‐assisted Savary–Gilliard bougie dilatation (7, 9, and 11 mm dilators) in a single session, with immediate improvement in swallowing and no procedural complications; Follow‐up at 2 weeks demonstrated sustained clinical improvement, and endoscopic follow‐up is planned.

**Conclusion:**

This case highlights the importance of considering inherited blistering disorders in children with chronic skin fragility and progressive dysphagia and illustrates that careful, staged bougie dilatation can safely relieve EB‐associated oesophageal stricture.

## 1. Introduction

Epidermolysis bullosa (EB) is a heterogeneous group of inherited mechano‐bullous disorders characterized by fragility of the skin and mucous membranes, leading to blister formation following minimal trauma, due to defects in structural proteins responsible for dermo‐epidermal adhesion [1, 2]. Blisters form below the basal keratinocytes at the dermo‐epidermal junction due to mutations in structural proteins (e.g., Type VII collagen, laminin‐332, and integrins) that weaken dermo‐epidermal adhesion [2, 3]. Mechanical trauma causes separation of the epidermis from the dermis, resulting in tense subepidermal blisters, often with scarring and mucosal involvement [2, 3]. Esophageal involvement in subepidermal EB results from repeated mucosal injury and healing with fibrosis, eventually leading to esophageal stricture formation [4]. Clinically, this may present as progressive dysphagia, feeding difficulties, malnutrition, and growth retardation, particularly in severe forms of the disease [4]. Esophageal strictures represent a challenging complication requiring multidisciplinary management.

This complication is well recognized in the dermatology literature but remains under‐reported in gastroenterology practice, where affected children may first present with dysphagia rather than overt skin disease [5]. We report a case of clinically suspected EB presenting with oesophageal stricture, successfully managed with Savary–Gilliard bougie dilatation.

## 2. Case Presentation

A 10‐year‐old girl from Chattogram, Bangladesh, presented with progressive dysphagia to solid foods of one‐and‐a‐half months duration. She had experienced recurrent bullous skin lesions since birth, healing with hypopigmented scarring and recurring after minor trauma; however, her skin disease had not previously been formally evaluated by a dermatologist or biopsied, and the present dysphagia was in fact the presenting complaint that prompted her first comprehensive diagnostic work‐up. She had previously undergone one unsuccessful attempt at oesophageal dilatation.

Her parents were healthy and nonconsanguineous. Her younger sister had similar blistering skin lesions but has not undergone formal dermatological evaluation or biopsy and has no reported feeding or swallowing difficulties.

**FIGURE 1:**
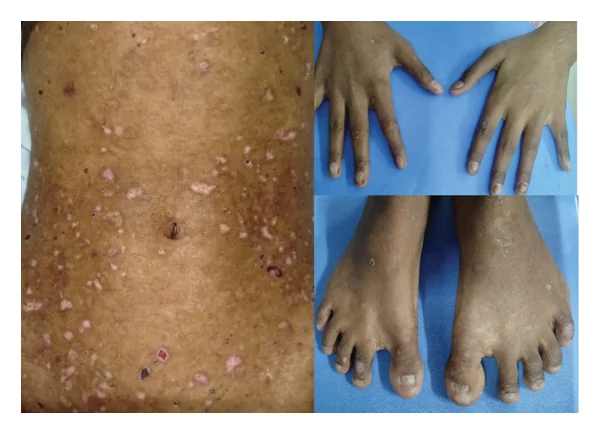
Clinical photographs of the trunk, hands, and feet showing multiple hypopigmented scars and incomplete, dystrophic nails.

On examination, she was malnourished and mildly anaemic. Multiple hypopigmented scars were present over the trunk, hands, and feet. Nail dystrophy and a smooth depapillated tongue were noted (Figure 1).

**FIGURE 2:**
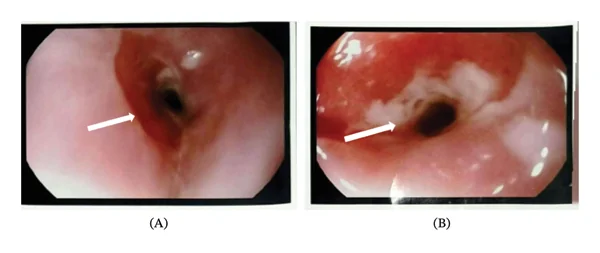
Upper gastrointestinal endoscopy demonstrating a 5‐cm‐long oesophageal stricture with superficial ulceration. (A) The proximal extent of the stricture beginning at 18 cm from the incisors, while (B) shows its distal extent at 22 cm from the incisors.

**FIGURE 3:**
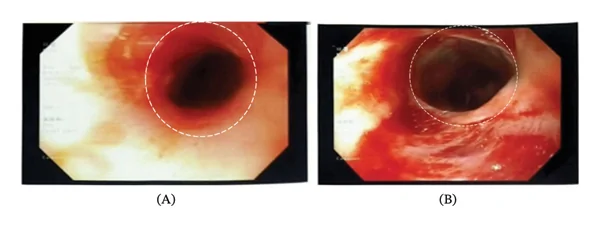
Upper gastrointestinal endoscopy showing the oesophageal stricture at 18 cm from the incisors before (A) and after (B) serial fluoroscopy‐guided Savary–Gilliard bougie dilatation (7, 9, and 11 mm), demonstrating improved luminal patency after the procedure.

**FIGURE 4:**
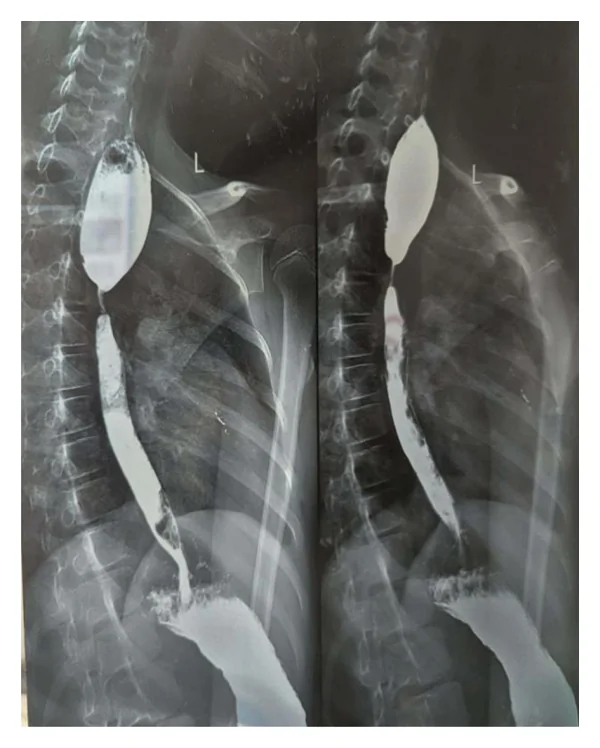
Barium swallow showing a tight upper thoracic oesophageal stricture with pre‐ and poststenotic dilatation.

Upper gastrointestinal endoscopy demonstrated oesophageal strictures from 18 cm to 22 cm from the incisors (combined stricture length approximately 5 cm), with superficial ulceration; the endoscope could not be advanced through the stricture (Figures 2 and 3). A barium swallow showed a tight upper thoracic oesophageal stricture with pre‐ and poststenotic dilatation (Figure 4). The patient had previously undergone an unsuccessful attempt at oesophageal dilatation and presented with progressive dysphagia requiring both diagnostic evaluation and potential therapeutic intervention. Therefore, upper gastrointestinal endoscopy was initially performed. Subsequently, a barium swallow study was obtained to better define the location, extent, and morphology of the nontraversable stricture and to assist procedural planning.

Skin biopsy and histopathological examination demonstrated a subepidermal vesiculobullous lesion with separation at the dermo‐epidermal junction accompanied by superficial perivascular eosinophilic and neutrophilic inflammatory infiltrates. Direct immunofluorescence was negative for IgA, IgG, and C3 deposition, making autoimmune subepidermal blistering disorders less likely.

Genetic testing, immunomapping, electron microscopy, and additional diagnostic immunofluorescence studies were unavailable.

Given the lifelong history of blistering skin disease, the family history, and histopathological evidence of a subepidermal blistering disorder, the patient was considered to have clinically suspected inherited EB, with the oesophageal stricture occurring in this context; a definitive causal or genetic link could not be established given the unavailability of confirmatory testing (see Table 1).

**TABLE 1:** Investigation profile.

Name of investigation	Patient’s value
CBC	Hb 10.6 g/dL, WBC 16k/cu mm, Neutrophils 74%, Lymphocyte 22%, Eosinophil 2%, MCH 27, MCHC 31.4, ESR 28
Total Eosinophil Count	160/cu mm
PBF	Mild normocytic normochromic anaemia with neutrophilic leukocytosis and thrombocytosis
RBS	83 mg/dL
S. Creatinine	0.5 mg/dL
S. Electrolyte	Na 139 mmol/L, K 4.6 mmol/L, Cl 99 mmol/L
Upper GI Endoscopy	Oesophageal strictures (18 cm–22 cm from incisors) with superficial ulceration; not traversable by the scope
Barium Swallow of Oesophagus	Upper thoracic tight stricture with pre‐ and poststenotic dilatation–chronic inflammatory
Echocardiogram	Normal, LVEF 60%, TAPSE 22 mm
Skin Biopsy with DIF	Subepidermal vesiculobullous disorder with eosinophils; DIF negative for IgA, IgG, and C3

*Note:* CBC, serum electrolytes, creatinine, and echocardiography were obtained as part of preanaesthetic assessment prior to endoscopic dilatation under sedation and were within normal limits. Genetic analysis was not possible due to limited resources.

### 2.1. Management and Outcome

Oesophageal strictures (18–22 cm from the incisors, combined length approximately 5 cm) were not traversable by the diagnostic endoscope. The patient underwent fluoroscopy‐guided, guidewire‐assisted oesophageal dilatation using Savary–Gilliard bougie dilators (7, 9, and 11 mm) under direct endoscopic visualization, performed as three sequential dilations in a single session. The procedure was completed without immediate complications. Following dilatation, dysphagia improved markedly, and the patient tolerated oral feeding. Topical antibiotic and corticosteroid therapy were prescribed for the skin lesions. Clinical follow‐up at one day showed no recurrence of dysphagia or procedure‐related complications.

Clinical follow‐up at 2 weeks demonstrated sustained clinical improvement. The patient tolerated a normal solid diet without dysphagia or odynophagia. No procedure‐related complications occurred, and repeat oesophageal dilatation was not required. She had gained approximately 0.5 kg in body weight since the procedure. Endoscopic follow‐up to assess luminal patency and the need for repeat dilatation is planned.

## 3. Discussion

This case illustrates two points relevant to gastroenterology practice. First, oesophageal stricture may be the complication that brings a child with previously unrecognized inherited blistering skin disease to medical attention: although this patient’s skin lesions had been present since birth, they had never been formally evaluated or biopsied until progressive dysphagia prompted a full diagnostic work‐up. This underscores the value of considering EB and arranging dermatological referral, in any child with chronic blistering or scarring skin lesions, even before mucosal complications develop. Esophageal stricture is a debilitating complication of EB which may present with dysphagia, odynophagia, vomiting, and malnutrition. EB patients may experience other complications like hiatus hernia, esophagitis, decreased or uncoordinated peristalsis, pseudodiverticula, esophageal atony, and shortening of the esophagus [6]. DEB patients may have limited mouth opening due to scarring and contracture of the corners of the mouth [7]. Our patient had a diagnosis of clinically suspected EB, and she suffered from various skin lesions with nail dystrophy and esophageal stricture with superficial ulcer. Many studies reported that dysphagia is present in two‐thirds of the EB patients, and almost 70% suffered from esophageal stricture in the first 25 years of life [8]. In a series of 22 EB patients with esophageal stricture undergoing stricture dilatation, the mean age of onset of dysphagia was 48 months, with the earliest age being 10 months. The average time between the onset of symptoms and dilatation was 33 months with a range of 1 month–10 years [9]. Dysphagia can be due to oropharyngeal blistering and desquamation commonly seen in severely affected DEB patients.

This may explain the delay in diagnosis [9].

Second, the strictures were located in the upper thoracic oesophagus, a recognized common site of involvement in EB [9]. Endoscopy could not traverse the stricture, and fluoroscopy‐guided, guidewire‐assisted Savary–Gilliard bougie dilatation produced immediate symptomatic improvement without complications. Savary Gillard dilatation is the preferred treatment for the esophageal stricture in EB patients, as it is safe and well tolerated, which is found evident in many studies [9–14]. Our patient had a single stricture; that is why it was dilated by bougie dilatation by fluoroscopy visualization. Although balloon dilatation is an accepted alternative, fluoroscopy‐guided, guidewire‐assisted Savary–Gilliard bougie dilatation was selected because it allowed controlled sequential dilatation of a long, tight upper oesophageal stricture. In addition, Savary dilators were readily available in our centre and represent the standard technique routinely used in our endoscopy unit. After dilatation of the esophagus, our patient’s symptoms of dysphagia and vomiting resolved instantly.

This case also illustrates the diagnostic challenges of confirming rare inherited skin disorders in resource‐limited settings, where genetic testing, immunomapping, and electron microscopy are not routinely available. In such settings, the clinical phenotype, family history, and histopathology remain the principal diagnostic tools, and the diagnosis should accordingly be framed as clinically suspected rather than definitively confirmed.

### 3.1. Limitations

Definitive confirmation and subtype classification of EB could not be established, as genetic testing, immunomapping, electron microscopy, and additional immunofluorescence studies were unavailable; the diagnosis therefore remains clinically suspected rather than confirmed, and the relationship between the skin disease and the oesophageal stricture, while consistent with recognized EB phenotypes, cannot be regarded as definitively causal. The younger sibling’s skin disease has not been formally evaluated or biopsy‐confirmed. Clinical follow‐up after dilatation was limited to one day; endoscopic follow‐up is planned but had not yet been performed at the time of this report.

## 4. Conclusion

Clinically suspected EB should be considered in children with chronic blistering skin disease and progressive dysphagia, particularly when the skin disease itself has not previously been diagnosed. Early recognition, dermatological referral, and timely endoscopic intervention may improve symptoms and nutritional status.

## Author Contributions

Mohammad Abu Faisal managed the patient, performed the endoscopic intervention, conceived the report, reviewed the literature, and drafted the manuscript. Sara Tasnim Mahpara contributed to patient management, data collection, literature review, and manuscript revision.

## Funding

This research received no external funding.

## Disclosure

The authors approved the final manuscript.

## Ethics Statement

Written informed consent for publication, including clinical, radiological, endoscopic, and pathology images, was obtained from the patient’s parent. Institutional ethics committee approval was not required for the case report as per the institutional review board of Chittagong Medical College.

## Conflicts of Interest

The authors declare no conflicts of interest.

## Data Availability

All data generated or analyzed during this case report are included in this published article. Additional information is available from the corresponding author upon reasonable request.

## References

[1] Fine J. D. , Bruckner-Tuderman L. , Eady R. A. J. et al., Inherited Epidermolysis Bullosa: Updated Recommendations on Diagnosis and Classification, Journal of the American Academy of Dermatology. (2014) 70, no. 6, 1103–1126, 10.1016/j.jaad.2014.01.903. 24690439

[2] Bolognia J. L. and Schaffer J. V. , Lorenzo Cerroni, Dermatology, 2017, 2, 4th edition, https://shop.elsevier.com/books/dermatology-2-volume-set/bolognia/978-0-7020-6275-9.

[3] Has C. , Nyström A. , Saeidian A. H. , Bruckner-Tuderman L. , and Uitto J. , Epidermolysis Bullosa: Molecular Pathology of Connective Tissue Components in the Cutaneous Basement Membrane Zone, Matrix Biol Journal Int Soc Matrix Biol. (2018) 71–72, 313–329, 10.1016/j.matbio.2018.04.001. 29627521

[4] Fine J. D. and Mellerio J. E. , Extracutaneous Manifestations and Complications of Inherited Epidermolysis Bullosa: Part II. Other Organs, Journal of the American Academy of Dermatology. (2009) 61, no. 3, 387–402, 10.1016/j.jaad.2009.03.053. 19700011

[5] El Hachem M. , Caldaro T. , Lara-Corrales I. et al., Management of Oesophageal Strictures in Inherited Epidermolysis Bullosa: A Clinical Practice Guideline, British Journal of Dermatology. (2025) 193, no. 3, 394–404, 10.1093/bjd/ljaf191. 40393138 PMC12666796

[6] Wong W. L. , Entwisle K. , and Pemberton J. , Gastrointestinal Manifestations in the Hallopeau-Siemens Variant of Recessive Dystrophic Epidermolysis Bullosa, British Journal of Radiology. (1993) 66, no. 789, 788–793, 10.1259/0007-1285-66-789-788. 8220949

[7] Marchili M. R. , Spina G. , Roversi M. et al., Epidermolysis Bullosa in Children: The Central Role of the Pediatrician, Orphanet Journal of Rare Diseases. (2022) 17, no. 1, 10.1186/s13023-021-02144-1. PMC8978425 35379269

[8] Gollu G. , Ergun E. , Ates U. , Can O. S. , and Dindar H. , Balloon Dilatation in Esophageal Strictures in Epidermolysis Bullosa and the Role of Anesthesia, Dis Esophagus Off Journal Int Soc Dis Esophagus. (2017) 30, no. 3, 1–6, 10.1111/dote.12503. 27133813

[9] Castillo R. O. , Davies Y. K. , Lin Y. C. , Garcia M. , and Young H. , Management of Esophageal Strictures in Children With Recessive Dystrophic Epidermolysis Bullosa, Journal of Pediatric Gastroenterology and Nutrition. (2002) 34, no. 5, 535–541, 10.1097/00005176-200205000-00012. 12050581

[10] Freeman E. B. , Köglmeier J. , Martinez A. E. et al., Gastrointestinal Complications of Epidermolysis Bullosa in Children, British Journal of Dermatology. (2008) 158, no. 6, 1308–1314, 10.1111/j.1365-2133.2008.08507.x. 18363753

[11] Azizkhan R. G. , Stehr W. , Cohen A. P. et al., Esophageal Strictures in Children With Recessive Dystrophic Epidermolysis Bullosa: An 11-Year Experience With Fluoroscopically Guided Balloon Dilatation, Journal of Pediatric Surgery. (2006) 41, no. 1, 55–60, 10.1016/j.jpedsurg.2005.10.007. 16410108

[12] De Angelis P. , Caldaro T. , Torroni F. et al., Esophageal Stenosis in Epidermolysis Bullosum: A Challenge for the Endoscopist, Journal of Pediatric Surgery. (2011) 46, no. 5, 842–847, 10.1016/j.jpedsurg.2011.02.017. 21616238

[13] Spiliopoulos S. , Sabharwal T. , Krokidis M. et al., Fluoroscopically Guided Dilation of Esophageal Strictures in Patients With Dystrophic Epidermolysis Bullosa: Long-Term Results, American Journal of Roentgenology. (2012) 199, no. 1, 208–212, 10.2214/AJR.11.8159. 22733914

[14] Mortell A. E. and Azizkhan R. G. , Epidermolysis Bullosa: Management of Esophageal Strictures and Enteric Access by Gastrostomy, Dermatologic Clinics. (2010) 28, no. 2, 311–318, 10.1016/j.det.2010.01.012. 20447496

